# Physiopathology of nonalcoholic fatty liver disease: from diet to nutrigenomics

**DOI:** 10.1097/MCO.0000000000000859

**Published:** 2022-08-04

**Authors:** Paola Meneghel, Elisa Pinto, Francesco Paolo Russo

**Affiliations:** Department of Surgery, Oncology and Gastroenterology, University Hospital Padua, Padova, Italy

**Keywords:** de-novo lipogenesis, Mediterranean diet, microbiota, NAFLD, saturated fatty acids

## Abstract

**Purpose of review:**

Nonalcoholic fatty liver disease (NAFLD) is the most common cause of chronic liver disease worldwide and is strongly associated with metabolic disorders, such as obesity, type 2 diabetes mellitus, and metabolic syndrome, to the extent that a new definition of metabolic associated fatty liver disease has been proposed.

**Recent findings:**

Insulin resistance, worsened by a high-fat and high-carbohydrate diet, is the key to the physiopathology of hepatic steatosis. This is driven by several mechanisms that are mostly activated at a genetic level, such as de-novo lipogenesis and triglyceride synthesis. Therefore, many diet regimens have been studied, although significant controversies remain regarding their metabolic effects and long-term sustainability.

**Summary:**

In this review, we summarized the role and effects of the main macronutrients on the development of NAFLD and discussed the molecular mechanisms involved. We also discussed the importance of genetic polymorphisms, epigenetic alterations, and dysbiosis to determine if lifestyle modification and a specific dietary regimen could be an essential part of NAFLD treatment.

## INTRODUCTION

Nonalcoholic Fatty Liver Disease (NAFLD) is characterized by excessive fat deposits in the liver and encompasses a broad spectrum of histologic conditions, ranging from simple hepatic steatosis to nonalcoholic steatohepatitis (NASH), NASH-related cirrhosis, and hepatocellular carcinoma.

NAFLD is the most common cause of chronic liver disease worldwide with an estimated global prevalence of ∼25% [1^▪^], and it is strongly associated with metabolic disorders, including obesity, type 2 diabetes mellitus (T2DM), and metabolic syndrome (MET).

Recently, a panel of international experts proposed “MAFLD” (i.e., metabolic associated fatty liver disease) as a new definition for the diagnosis of hepatic steatosis besides overweight/obesity, presence of T2DM, or the evidence of metabolic dysregulation, to highlight the importance of metabolically unhealthy patterns that drive the hepatic disease and differentiate from the past definition of NAFLD [2^▪▪^].

The key to the physiopathology of hepatic fat accumulation is insulin resistance, a condition that is characterized by poor tissue response to insulin. The overabundance of circulating fatty acids derived from adipose tissue and excess dietary intake accumulate in the liver, wherein de-novo lipogenesis (DNL) also occurs under conditions of hyperglycemia and hyperinsulinemia.

The treatment of NAFLD patients relies on lifestyle modifications, with the necessity to lose ≥7–10% of the initial body weight through diet and exercise, because lower weight is associated with improvement in liver histology and liver enzyme levels [3^▪^]. The lack of rigorous prospective trials comparing various diets in NAFLD has prevented the American Gastroenterological Association from adding a specific macronutrient diet recommendation [4]. Yet, current European guidelines [5^▪▪^] and multisociety Italian guidelines [6] suggest adherence to the Mediterranean diet because it showed beneficial effects both on liver histology and on some cardiovascular parameters [7]. In this review, we discussed the role of the major players in the development of NAFLD. 

**Box 1:**
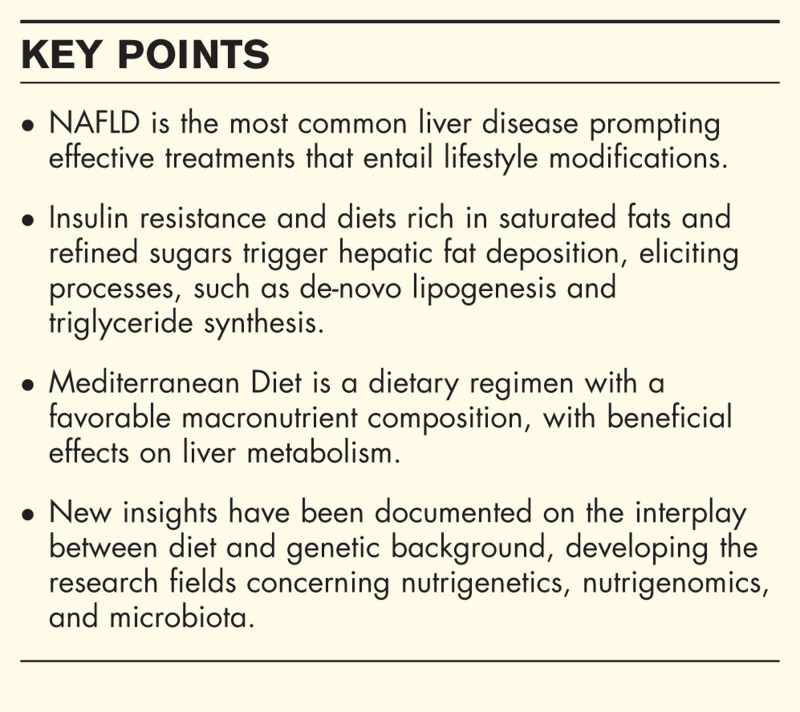
no caption available

## LIPIDS

Fatty acids include saturated fatty acids (SFAs), monounsaturated fatty acids (MUFAs), and polyunsaturated fatty acids (PUFAs). Among the unsaturated fatty acids, linoleic acid and α-linolenic acid are essential to humans because they cannot be synthesized *ex novo*, and dietary intake is their only source. Their 18-carbon chain contains a special double bond in a specific position: 18 : 2 n-6 for linoleic acid and 18 : 3 n-3 for α-linolenic acid. Starting from here, the chains are elongated to build omega-6 (n-6) and omega-3 (n-3) PUFAs.

As stated in previous reviews [8,9], Western diets are rich in SFAs, found in meat and dairy products, and poor in PUFAs (found in fish, seeds, nuts, and vegetable oils) and in MUFAs (found in olive oil). SFAs are associated with an increase in liver fat deposition, driving insulin resistance, lipolysis in adipose tissue and triglyceride synthesis in the liver. On the contrary, PUFAs drive an opposite process, i.e., a reduction in liver fat content, as shown by imaging methods, and a decrease in liver enzymes and steatosis scores.

The intake of excessive SFAs is associated with hepatic steatosis and might lead to hepatocytic damage by altering mitochondrial structure and function. Despite an increase in mitochondrial respiration, the respiratory transport chain appears dysfunctional and inefficient, with Reactive Oxygen Species production that damages the cellular structures causing inflammation, apoptosis, and scarring of the liver. Additionally, SFAs alter the composition of mitochondrial membranes [10]. Previous studies hypothesized that they exert an effect at a genomic level by stimulating liver PGC-1beta (PPAR-gamma coactivator-1 beta) and Sterol Regulatory Element-binding Protein 1c; thus, eliciting the expression of some genes involved in DNL (Stearoyl-CoA desaturase-1, FAS, and Diacylglycerol Acyltransferase) [11].

PUFAs help to revert hepatic steatosis. An interventional study investigated liver fat accumulation after overfeeding 39 normal-weight individuals with muffins rich in SFAs (palm oil) or n-6 PUFAs (sunflower oil) for 7 weeks. The individuals showed a similar weight gain, but considerably higher liver fat and a twofold increase in visceral adipose tissue were associated with SFAs, as assessed by magnetic resonance imaging. Conversely, PUFAs caused an increase in lean tissue, but its intake has changed over the years [12]. As previously reported in the review by Yki-Järvinen *et al.* that summarized the results of a few interventional studies, hyper- and iso-caloric SFA-enriched diets significantly increased intrahepatic triglyceride content in lean and obese patients by increasing adipose tissue lipolysis and therefore causing an augmentation of triglyceride synthesis in the liver [13^▪▪^]. The same review documented that PUFAs do not sort the same effect on IHTG and liver histology [13^▪▪^]. Another recent review compared the outcomes of 22 randomized controlled trials (RCTs) that investigated the effects of PUFAs on liver histology, supporting its supplementation in NAFLD. N-3 PUFAs supplementation significantly reduced liver fat compared with placebo (pooled risk ratio 1.52; 95% confidence interval, 1.09–2.13) and in addition to that sorted positive effects on other serum and anthropometric parameters, such as levels of triglyceride, total cholesterol, high-density lipoprotein, and body-mass index [13^▪▪^].

A few RCTs confirmed these results, demonstrating that n-3 supplementation in the diet had a beneficial effect on liver fat, but no did not improve NASH features and fibrosis [14].

Interestingly, a low n-6/n-3 ratio is required to benefit from PUFAs. Patients affected by NAFLD display a lower consumption of n-3 PUFAs and a higher n-6/n-3 ratio intake [15], which may be related to an altered linoleic acid/α-linolenic acid ratio and pathological metabolism of pro-inflammatory PUFAs that can enhance the production of inflammatory mediators [8]. Thus, NAFLD patients need to maintain a low n-6/n-3 intake to take advantage of n-3 PUFAs [16].

## CARBOHYDRATES

Carbohydrates may be classified as simple sugars and complex sugars, and among these, glucose, fructose, and high fructose corn syrup (HFCS) play a key role in metabolic liver disease [17,18].

High carbohydrate intake leads to an increase in DNL and the accumulation of triglyceride in hepatocytes through the activation of transcription factors like Carbohydrate Response Element Binding Protein and genes, such as PPARγ coactivator 1b (peroxisome proliferator-activated receptor gamma coactivator 1-beta) and Liver X Receptor, involved in hepatic lipogenesis [19,20^▪▪^]. The glucose that is not converted to glycogen in the muscles is directed to the liver, leading to a compensatory increase in insulin levels and the onset of progressive insulin resistance. The latter initiates hepatic steatosis by providing precursors and substrates for DNL and mitochondrial β-oxidation.

Fructose, on the contrary, which is present both in natural and artificially sweetened foods, is more directly implicated in the development of NAFLD because its metabolism occurs primarily in the liver, where it bypasses the rate-limiting step of glycolysis at the level of phosphofructokinase, which causes most metabolic disorders [21]. The dietary intake of added fructose, specifically in the form of sucrose or HFCS, was found to be positively correlated with NAFLD [22].

By reducing beta-oxidation, fructose enhances the mechanism of insulin resistance (like glucose); thus, increasing DNL [23]. Fructose metabolism also increases uric acid, which leads to mitochondrial dysfunction and the production of ROS, resulting in oxidative stress.

Moreover, a diet rich in carbohydrates, such as sucrose and fructose, can also lead to epigenetic alterations, such as hypermethylation and hypomethylation, that may directly alter the expression of genes involved in lipid metabolism, including those that increase hepatic fat accumulation or reduce hepatic fat removal [24,25]. Finally, as reported later, an excess of carbohydrates affects the homeostasis of the intestinal microbiota, contributing to the development of NAFLD [26].

## MEDITERRANEAN DIET AND OTHERS

MAFLD patients usually follow Western diets that tend to be hypercaloric and rich in processed foods, salt, added sugars, and red meat, and poor in whole grains, legumes, fruits, and vegetables.

As previously stated, European Association for the Study of the Liver guidelines recommend diets with a macronutrient composition that resembles the Mediterranean diet, which is based on a high intake of fresh vegetables and fruits, cereals, legumes, olive oil, and nuts, moderate intake of fish and poultry, and low consumption of red meat and dairy products. The evidence supporting its implementation was well described in a recent Italian review [7]. Mediterranean diet is a high-fat diet (35%–45% of total daily energy intake) with a lower proportion of SFAs and cholesterol, and a higher proportion of MUFAs and PUFAs with a balanced omega-6 to omega-3 ratio. Carbohydrates comprises of 35%–40% of the daily energy intake (mostly in the form of complex carbohydrates with a poor presence of simple sugars), whereas proteins constitute the remaining 15%–20% of the daily energy intake. Additionally, Mediterranean diet is rich in whole grains and fibers, which favor prebiotic effects on gut homeostasis. Many interventional studies have been conducted in the past 20 years to determine the effects of Mediterranean diet on NAFLD patients. In a trial involving 50 overweight patients with fatty liver, the patients were randomized to Mediterranean diet, Mediterranean diet + antioxidant complex oral supplement, or control. Both groups assigned to Mediterranean diet showed a significant reduction in hepatic fat content, with a greater effect on anthropometric parameters and insulin sensitivity in subjects that were also administered antioxidant supplementation. These results showed the important pathogenetic role of oxidative stress in MAFLD physiopathology [27]. Moreover, Mediterranean diet showed beneficial effects on ameliorating insulin sensitivity as summarized in the review by Hashem *et al.*[28].

Additionally, another recent trial suggested a new strategy of green-Mediterranean diet, amplified with green plant and polyphenols, such as Mankai, green tea, and walnuts, which demonstrated double intrahepatic fat loss, relative to other healthy nutritional strategies, on magnetic resonance spectroscopy (MRS) [29]. On the other hand, in an RCT in which patients were assigned to isocaloric Mediterranean diet or a low-fat diet, liver fat reduction after the intervention was similar in both groups, determined through MRS. Additionally, liver enzyme levels were also similar, although the Mediterranean diet had an impact on cardiometabolic risk factors (Framingham Risk Score, total cholesterol, serum triglyceride, and HB1Ac). Moreover, the polyphenol oleocanthal extracted from extra virgin olive oil, which is a part of the Mediterranean diet, has antifibrotic effects on the liver through the upregulation of pro-fibrotic and inflammatory genes and the downregulation of pro-fibrotic miRNAs [30].

Thus, studies have shown that the Mediterranean diet is effective in reverting the severity of hepatic steatosis, although most studies rely on radiological or serological endpoints and lack histologic confirmation via liver biopsy. Ketogenic diet, dietary approach to stop hypertension, and intermittent fasting have been tested in small cohorts of NAFLD patients [7]. The results showed efficacious weight loss in all tested regimens, although confirmed outcomes on liver histology were not documented. Therefore, these diets can be recommended for weight loss, which is the best therapeutic approach to MAFLD. However, strong comparisons among the different regimens cannot be made.

## NUTRIGENETICS AND NUTRIGENOMICS

The contribution of the genetic background of an individual and the interplay between the genetic background and nutritional habits in the onset and worsening of MAFLD is an emerging field of research known as nutrigenetics and nutrigenomics [8]. PNPLA3, MBOAT7, and TM6SF2 are the genetic variants that have shown the greatest association with MAFLD pathogenesis.

The rs738409 SNP variant of the PNPLA3 gene encodes a dysfunctional form of a transmembrane protein localized on the endoplasmic reticulum of hepatocytes and adipocytes that, in case of insulin resistance and obesity, cannot exert its triglyceride hydrolase activity, causing an imbalance in cellular homeostasis and the accumulation of lipid droplets. Moreover, this genetic variant is responsible for the activation of HSCs and the promotion of pro-inflammatory and pro-fibrotic responses. Regarding the response to specific nutrients, when this variant is present, a minor hydrolase activity occurs with the consequent overload of n-6 proinflammatory-derived species (e.g., AA) that promotes NASH [8].

In the presence of the rs641738 variant of the MBOAT7 gene, a decrease in the expression of a membrane-bound enzyme that incorporates unsaturated FAs (and preferentially AA) in phosphatidylinositol molecule occurs, which impairs several cellular pathways and causes steatosis, inflammation, and fibrosis. In hyperinsulinemia and after a high intake of saturated fats, the impaired function of the receptors becomes even more relevant, promoting the synthesis of saturated phospholipids and fat deposition in hepatocytes [8].

The E167K variant of the TM6SF2 gene also contributes to hepatic steatosis since it encodes a protein localized in the endoplasmic reticulum and Golgi apparatus that regulates triglyceride secretion. In the subjects presenting this variant, this latter process results impaired, contributing to intracellular triglyceride retention and thus to NAFLD. In conditions of high dietary assumption of fats and carbohydrates the effect of lipidic retention in hepatocytes is further increased [8].

## MICROBIOTA

The mechanisms underlying the development of NAFLD/NASH are multifactorial, and one such factor is the relationship between diet and intestinal microbiota [31^▪^,^26^].

Gut microbiota consists mainly of the following bacterial phyla: firmicutes, bacteroidetes, and actinobacteria. The concentration of these may vary depending on internal and external factors. Some studies have shown that a diet based mainly on animal by-products reduces beneficial bacterial microbiota and increases opportunistic bacterial microbiota, lipopolysaccharides, short-chain fatty acids, and inflammatory cytokines [26,32]. This increases the risk of chronic diseases, including METs and NAFLD, whereas a plant product-based diet reduces this risk [33]. In general, a high-fat, animal-based diet leads to dysbiosis that causes metabolic and immunological alterations, such as insulin resistance [34,35], and the production of ROS through the consumption of glycine, the precursor of glutathione [36^▪▪^]. From an immunological perspective, mucosal immunity and intraepithelial lymphocytes decrease and inflammatory chemokines increase simultaneously via the TLR4 signaling pathway [37–39]. Dysbiosis also alters the integrity of the intestinal barrier, causing translocation of bacteria and bacterial metabolites to the liver via the portal vein [31^▪^,^40^]. Altered microbial microbiota may also alter the production of bile acids, which influence the development of NAFLD and the progression of the disease to NASH [41]. The study on the correlation between diet, microbiota, and NAFLD might help to develop new methods of prevention and treatment of NAFLD by proposing a healthy and defined diet, along with the use of probiotics that are effective in the treatment of NAFLD.

## CONCLUSION

Information on NAFLD and the nutrients affecting its pathophysiology suggest reducing the content of saturated fat, refined carbohydrates, red/processed meats, and take-away foods in the diet and increasing the consumption of plant-based foods, along with energy restrictions and increasing weight loss, since this type of dietary regimen shows beneficial effects on liver histology by suppressing abnormal mechanisms that drive NAFLD. Future studies need to assess specific dietary models that affect liver histology and the composition of the microbiota.

## Acknowledgements

*None.*

### Financial support and sponsorship

*None.*

### Conflicts of interest

*There are no conflicts of interest.*

## REFERENCES AND RECOMMENDED READING

Papers of particular interest, published within the annual period of review, have been highlighted as:

▪ of special interest
▪▪ of outstanding interest
